# A 10-year review of primary major salivary gland cancers

**DOI:** 10.3332/ecancer.2020.1055

**Published:** 2020-06-12

**Authors:** Andreia Cruz, Helena Magalhães, Filipa Ferreira Pereira, José Dinis, Cláudia Vieira

**Affiliations:** 1 Medical Oncology Department, Instituto Português de Oncologia do Porto, Porto, Portugal; 2 Medical Oncology Department, Unidade Local de Saúde de Matosinhos – Hospital Pedro Hispano, Matosinhos, Portugal

**Keywords:** head and neck cancer, salivary gland cancer, parotid gland

## Abstract

Primary salivary gland cancers comprise a heterogeneous group of histological entities and represent less than 5% of head and neck malignancies. Surgical resection is the main treatment, and adjuvant radiotherapy is performed in selected cases. Chemotherapy is an option in metastatic or recurrent disease, with poor evidence. We aimed to review a 10-year experience of a cancer centre on major salivary gland cancers, focusing on clinical, pathological, treatment and patients’ outcomes data.

A total of 93 patients were identified, median age at diagnosis was 64 years (IQR, 23), and 51.6% were male. The parotid gland was the site of origin in 76.3% of cases. The most frequent histological type was salivary duct carcinoma (21.5%). All patients were submitted to surgery and adjuvant radiotherapy was performed in 74.2%. From 26 patients diagnosed with metastatic disease, 9 were treated with systemic therapy. At 8 years, disease-free survival was 54.6% and overall survival was 48.4%. Male sex, salivary duct carcinoma, stage pT3-4, stage pN2-3, high histologic grade, lymphovascular invasion and perineural invasion were negative prognostic indicators for disease-free survival and overall survival. Extracapsular spread was a negative prognostic indicator for overall survival. In the multivariable analysis, histological type—salivary duct carcinoma—kept significant negative impact in disease-free survival and high histologic grade in overall survival.

The most frequent histological type was salivary duct carcinoma, which is estimated to represent only 9% of salivary tumours. Patients with salivary duct carcinoma relapsed more than other histological types. High histologic grade was a negative prognostic indicator for overall survival.

## Background

Primary salivary gland cancers (SGC) are a rare and highly diverse group of tumours regarding histology, clinical behaviour and responsiveness to therapy. The incidence of SGC is estimated to be 1.31/100,000/year, representing less than 5% of all head and neck malignancies, with equal sex distribution and average age of 47 years [1, 2].

The 2017 World Health Organization classification comprises more than 20 different malignant histologies for SGC, with specific features and outcomes. The impact of genetic alterations is increasing, despite restricted to diagnostic purposes so far [2]. Mucoepidermoid carcinoma is the most frequent primary salivary malignancy, followed by adenoid cystic and acinic cell carcinoma [3]. Parotid gland is the most frequent site of origin, accounting for 70% of cases, with only 10% to 15% arising in minor salivary glands. Nevertheless, malignancy rate has been described as only 25% for parotid neoplasms, compared to 82% for minor salivary glands and 43% for submandibular glands [4].

It is recognised that risk factors for SGC include viruses, radiation exposure and occupation. Prognostic factors include stage—tumour size or extent (T), nodal status and extranodal extension (N) and presence of distant metastases (M) —, location, histological type and grade [5, 6]. Also, perineural and lymphovascular invasion, and even male sex have been associated with poorer outcomes [7, 8].

Currently, upfront surgical resection remains the standard of care for locoregional disease, with elective neck dissection according to histology and clinical stage [9, 10]. Adjuvant radiotherapy can be performed in high grade, advanced stage disease or incomplete resection [11], apparently with no benefit from concurrent chemotherapy [12]. Chemotherapy is an option in metastatic or recurrent setting, with CAP regimen (cyclophosphamide, adriamycin and cisplatin) having the greatest amount of data, despite no clear benefit on survival or over single-agent therapy [13, 14]. Hormonal therapy has shown activity in androgen-receptor positive SGC, which is detected in various histologies and in the majority of cases of salivary duct carcinoma (SDC) [15]. Also, there has been an increasing interest in defining routes to targeted therapies, with some preliminary evidence [16, 17].

In this study, we aim to review the incidence, pathology, clinical behaviour and outcomes in a cohort of patients with major SGC treated at a tertiary cancer centre over a 10-year period from 2006 to 2016.

## Methods

A retrospective cohort of patients with histological diagnosis of primary major SGC, admitted to a single tertiary cancer centre from March 2006 to July 2016, was evaluated.

Demographic and clinical-pathological features analysis was performed using descriptive statistics. Data were retrieved from patients’ charts regarding demographics, presentation, pathology, treatment and outcomes, including disease-free survival (DFS) and overall survival (OS). DFS and OS were estimated by the Kaplan–Meier method and compared by Cox regression. DFS was defined as time between curative treatment and local or distant recurrence; OS was defined as time between diagnosis and death from any cause. Follow-up was updated as of 31th October, 2019.

Statistical analysis was performed using SPSS Statistics for MacOS, version 25.0 (SPSS, Chicago, IL). Statistical significance was set at *p* < 0.05.

The study was conducted in accordance with the Declaration of Helsinki on biomedical research involving human subjects, and after the written approval of institutional ethics committee.

## Results

Between March 2006 and July 2016, 933 patients were diagnosed with salivary gland lesions in our institution, whose 32.9% (*n* = 307) had malignant histologies, and 10.0% (*n* = 93) were primary tumours. Median follow-up was 8 years (CI 95% 6.2–9.6). Demographics, clinical-pathological and treatment features are summarised in Table 1. Most patients were male and median age at diagnosis was 64 years (IQR, 23). The location of the primary tumour was mostly in parotid gland (76.3%, *n* = 71), followed by submandibular gland (22.6%, *n* = 21). The most frequent histological type was SDC (21.5%, *n* = 20), followed by adenoid cystic carcinoma (16.1%, *n* = 15) and mucoepidermoid carcinoma (14.0%, *n* = 13). Six cases of SDC (30%) arose in pleomorphic adenoma. All patients were submitted to surgery and most of them underwent adjuvant radiotherapy (74.2%, *n* = 69), in three cases with concurrent chemotherapy with cisplatin. A large number of patients were diagnosed with pT3 or pT4 disease (36.6%, *n* = 34) and with positive lymph nodes (18.3%, *n* = 17) although an important number of patients (31.2%, *n* = 29) had no lymph nodes removed (stage pNx). Eight patients were initially diagnosed with distant metastasis. Histological grade was classified as high in 24.7% (*n* = 23) of patients. Lymphovascular invasion was seen in 22.6% of patients (*n* = 21), perineural invasion in 37.6% (*n* = 35), carcinoma ex pleomorphic adenoma in 6.5% (*n* = 6) and extracapsular spread in 9.7% (*n* = 9). Facial nerve sacrifice during surgery was used as a surrogate for preoperative facial nerve involvement, and was present in 23.9% (*n* = 17) of patients who underwent parotidectomy. *ERBB2* and androgen receptor positivity was seen in two and six patients, from six and 11 analysed, respectively.

Distant metastasis at diagnosis were mainly seen in lung (four patients), but also in brain, bone, liver and skin, in one patient each. During follow-up, 34.1% (*n* = 29) of patients recurred, mainly locally (37.9%, *n* = 11), but also in lung (17.2%, *n* = 5), bone (10.3%, *n* = 3), liver (10.3%, *n* = 3), brain (3.4%, *n* = 1) and multiple sites (20.7%, *n* = 6). In the metastatic setting, nine patients were treated with systemic therapy, and chemotherapy regimens used were CAP and carboplatin with paclitaxel; one patient was treated with methotrexate in third line; and the patient with small cell carcinoma was treated with carboplatin and etoposide.

**Figure 1.**
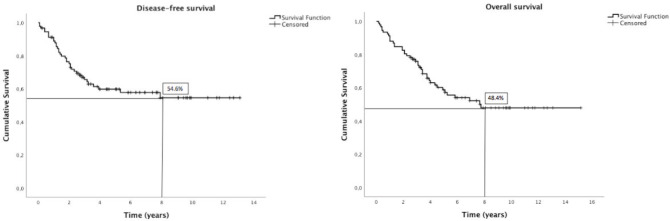
Kaplan–Meier plots of disease-free and overall survival.

At 8 years, DFS was 54.6% and OS was 48.4% (Figure 1). The results of univariate Kaplan–Meier survival analyses are summarised in Table 2, and multivariable survival models in Table 3. In the univariate analysis, male sex, SDC, stage pT3-4, stage pN2-3, high histologic grade, lymphovascular invasion and perineural invasion were significant negative prognostic indicators for DFS and OS. Extracapsular spread was a significant negative prognostic indicator for OS, but not for DFS. In the multivariable analysis just histological type—SDC—kept significant negative impact in DFS, but not in OS; and high histologic grade in OS, but not in DFS.

## Discussion

Primary SGC are rare and heterogeneous, and there is also some geographic variation in the frequency of tumour types, resulting in poorly documented epidemiology [2]. The incidence of major SGC is increasing, mainly parotid and smaller tumours, with regional and distant metastasis, probably due to earlier diagnosis and improved clinical staging. Aetiology and risk factors are not well established, thus the reason for this rising incidence is not well known [18]. Since Portugal is a small country accounting, approximately, 10 million inhabitants, and our institution is the biggest in the country dedicated to the treatment of cancer, we present, probably, the largest national series of major SGC patients.

In our cohort, the most frequent histological type was SDC, which is estimated to represent only 9% of salivary tumours [2], probably because our institution is a high-specialised oncology centre. Kleinsasser *et al* [19] described SDC in 1968, as an aggressive adenocarcinoma which resembles high-grade breast ductal carcinoma. This entity has been increasingly diagnosed, and there is currently a growing interest from the scientific community, with multiple publications of institutional series [20–30]. Actually, SDC is characterised by a very aggressive behaviour, with high rates of local or distant recurrence and tumour-related death [31, 32]. This is mostly diagnosed in elderly men, predominantly in the parotid gland. Pain, facial palsy and presence of calcifications on computed tomography scans are suggestive features of SDC. Node metastasis, lymphovascular and perineural invasion, and positive surgical margins have been described as adverse prognostic factors [31, 32]. SDC frequently overexpresses *EGFR* and *ERBB2*, but it is not clear whether that expression is associated with poor prognosis, or the role of targeted therapy [33]. Trastuzumab, an inhibitor of *ERBB2*, has been used for systemic therapy in patients with advanced SDC with promising responses [34–37]. SDC is also associated with androgen-receptor positivity in 67%–96% of cases, and, recently, a prospective phase II study showed that combined androgen blockade had equivalent efficacy and less toxicity for patients with androgen-receptor positive recurrent/metastatic or unresectable locally advanced SGC compared with conventional chemotherapy, which warrants further investigation [38–40]. Even PD-L1 expression and its correlation with survival and other promising therapeutic targets are under investigation in SDC and other histologies of SGC [41, 42].

In our cohort, patients with SDC relapsed more, and high histologic grade was a negative prognostic indicator for overall survival, which is in accordance with previous data. Only few patients were treated with palliative chemotherapy, due to its modesty activity. Data on the role of systemic therapy in the management of SGC is limited, and chemotherapy is generally reserved for advanced disease, when it is not suitable to surgery and/or radiation [13]. A systematic review of published data in the management of metastatic or locally recurrent adenoid cystic SGC demonstrated major objective responses in nine of 36 patients treated with CAP regimen (response rate 25%, 95% CI 11%–39%), but even in just four trials analysed, doses were different [43]. We had an adenoid cystic SGC patient with almost 3 years of progression-free survival after first line chemotherapy with CAP regimen for lung recurrence (500/50/50 mg/m^2^, q21d, six cycles). Despite the lack of robust data to support carboplatin-based regimens, we used it in combination with paclitaxel [44, 45], with manageable toxicity profile and progression-free survival between two and 28 months. A small number of patients were submitted to *ERBB2* and androgen receptor status determination and none was treated with targeted therapy, despite the positivity rate of 33.3% and 54.5%, respectively.

The present study was limited by its retrospective design and a small number of patients included, with multiple histologies. Prospective clinical trials, including those analysing the efficacy of adjuvant therapy and targeted therapy, are warranted. Priority should be given, also, to the molecular understanding of these tumours. Nevertheless, due to SGC rarity, such studies will be difficult to perform, and we must start to analyse what we have now, to aggregate multicenter data and set priorities for the future.

## Conclusions

In our cohort, the most frequent histological type was SDC, which is estimated to represent only 9% of salivary tumours [2]. Patients with SDC relapsed more, and high histologic grade was a negative prognostic indicator for overall survival. Only a few patients were treated with palliative chemotherapy, mainly with CAP regimen or combined carboplatin and paclitaxel.

## Conflicts of interest

The authors declare no conflicts of interest

## Funding statement

There was no funding for this study.

## Figures and Tables

**Table 1.** Demographics, clinical-pathological and treatment features.

Characteristics		*n*	%
Sex	Male Female	48 45	51.6 48.4
Age	Median, years Interquartile Range, years	64 23	
Eastern Cooperative Oncology Group (ECOG) performance status	0 1 2 3 Missing	21 5 1 1 65	22.6 5.4 1.1 1.1 65.9
Primary site	Parotid gland Submandibular gland Sublingual gland	71 21 1	76.3 22.6 1.1
Histological type	Salivary duct carcinoma Adenoid cystic carcinoma Mucoepidermoid carcinoma Myoepithelial carcinoma Acinic cell carcinoma Carcinoma ex pleomorphic adenoma Squamous cell carcinoma Oncocytic carcinoma Basal cell adenocarcinoma Adenocarcinoma, not otherwise specified Small cell carcinoma Carcinosarcoma Low-grade cribriform cystadenocarcinoma Solitary fibrous tumour	20 15 13 11 9 6 6 3 3 3 1 1 1 1	21.5 16.1 14.0 11.8 9.7 6.5 6.5 3.2 3.2 3.2 1.1 1.1 1.1 1.1
Stage T	pT1 pT2 pT3 pT4 Missing	26 27 22 12 6	28.0 29.0 23.7 12.9 6.5
Stage N	pN0 pN1 pN2 pN3 pNx Missing	41 6 8 3 29 6	44.1 6.5 8.6 3.2 31.2 6.5
Stage M	cM0 cM1 Missing	82 8 3	88.2 8.6 3.2
Grade	High Intermediate Low Missing	23 8 17 45	24.7 8.6 18.3 48.4
Lymphovascular invasion	Yes No Missing	21 48 24	22.6 51.6 25.8
Perineural invasion	Yes No Missing	35 37 21	37.6 39.8 22.6
Carcinoma ex pleomorphic	Yes No Missing	6 55 32	6.5 59.1 34.4
Extracapsular spread	Yes No Missing	9 52 32	9.7 55.9 34.4
Facial nerve sacrifice	Yes No Missing	17 45 9	23.9 63.4 12.7
*ERBB2* status	Positive Negative Missing	2 4 87	2.2 4.3 93.5
Androgen receptor status	Positive Negative Missing	6 5 82	6.5 5.4 88.2
Local treatment	Surgery Adjuvant radiotherapy Adjuvant chemoradiotherapy	93 66 3	100 71 3.2
Systemic treatment	Adjuvant chemotherapy Palliative chemotherapy	1 9	1.1 9.7

**Table 2.** Univariate analysis of DFS and OS.

Characteristic		DFS, years		OS, years	
		Median	*p*	Median	*p*
Sex	Male Female	3.0 NR	0.015	4.9 NR	0.047
Age, years	<65 ≥65	NR 7.9	0.225	NR 4.3	0.098
Primary site	Parotid gland Other	NR NR	0.990	7.7 NR	0.510
Histological type	Salivary duct carcinoma Other	1.8 NR	<0.001	3.1 NR	<0.001
Stage T	pT1-2 pT3-4	NR 3.2	0.023	NR 3.4	0.001
Stage N	pN0-1 pN2-3	NR 2.8	<0.001	7.8 2.5	<0.001
Grade	High Intermediate/Low	1.8 NR	<0.001	3.1 NR	<0.001
Lymphovascular invasion	Yes No	2.0 NR	0.001	3.2 NR	0.001
Perineural invasion	Yes No	3.6 NR	0.028	4.0 NR	0.004
Carcinoma ex pleomorphic	Yes No	NR NR	0.283	4.9 7.7	0.322
Extracapsular spread	Yes No	3.0 NR	0.094	2.5 NR	<0.001
Facial nerve sacrifice	Yes No	3.2 NR	0.613	4.9 NR	0.509
*ERBB2* status	Positive Negative	1.8 1.3	0.782	5.1 3.2	0.317
Androgen receptor status	Positive Negative	1.8 NR	0.682	3.2 5.3	0.578
NR—not reached.					

**Table 3.** Multivariable analysis of DFS and OS.

Characteristics		DFS		OS	
		HR (95% CI)	p	HR (95% CI)	p
Sex	Male Female	36.4 (0.9-1473.6)	0.057	3.2 (0.4-26.4)	0.28
Histological type	Salivary duct carcinoma Other	43.5 (1.1-1686.1)	0.043	6.1 (0.2-201.1)	0.310
Stage T	pT1-2 pT3-4	0.72 (0.06-8.7)	0.800	0.05 (0.0-2.4)	0.132
Stage N	pN0-1 pN2-3	0.45 (0.02-11.1)	0.624	9.6 (0.1-1492.9)	0.379
Grade	High Intermediate/Low	0.0 (0.0-6965.0)	0.936	0.02 (0.001-0.9)	0.044
Lymphovascular invasion	Yes No	0.13 (0.01-1.8)	0.129	0.35 (0.02-6.9)	0.488
Perineural invasion	Yes No	7.4 (0.4-127.9)	0.167	0.57 (0.06-5.3)	0.623
Extracapsular spread	Yes No	NA		0.02 (0.0-1.5)	0.075
NA—not applicable.					
